# The Feasibility and Acceptability of Sharing Video Recordings of Amyotrophic Lateral Sclerosis Clinical Encounters With Patients and Their Caregivers: Pilot Randomized Clinical Trial

**DOI:** 10.2196/57519

**Published:** 2024-06-26

**Authors:** Reed W R Bratches, Jeffrey Cohen, Elizabeth Carpenter-Song, Lisa Mistler, Paul J Barr

**Affiliations:** 1 School of Nursing University of Alabama at Birmingham Birmingham, AL United States; 2 Dartmouth College Hanover, NH United States

**Keywords:** feasibility, acceptability, amyotrophic lateral sclerosis, digital intervention, ALS, video recording

## Abstract

**Background:**

Multidisciplinary clinics (MDCs) provide benefits to patients with amyotrophic lateral sclerosis (ALS) and their caregivers, but MDC visits are information-heavy and can last 4 hours, with patients and caregivers meeting with multiple specialists within each MDC visit. There are questions about the effectiveness of current methods of sharing information from MDCs with patients. Video recordings are a promising new method of sharing information that may allow patients and caregivers to revisit the MDC and remind them of clinical recommendations and conversations.

**Objective:**

The objective of this trial is to determine the feasibility and acceptability of sharing information through video recordings of ALS MDC visits with patients and caregivers.

**Methods:**

This study was a randomized, controlled pilot trial with 3 months of follow-up from April 2021 to March 2022 in a rural multidisciplinary neurology clinic. We recruited patients with ALS, their caregivers, and their clinicians. Patients and their caregivers were randomized to either receive their normal after-visit summary (treatment as usual) or to receive their normal after-visit summary and a video recording of their MDC visit (video). Each specialist visit had its own recording and was accessible by patients and caregivers using a secure web-based platform called HealthPAL over a 3-month follow-up period. Primary study outcomes were feasibility and acceptability of the video intervention measured by recruitment rate (target: 70%), percentage of participants watching videos (target: 75%), and the Feasibility of Intervention Measure and Acceptability of Intervention Measure (targets: 3/5). We hypothesized that video recording would be feasible and acceptable to patients and their caregivers.

**Results:**

Of the 30 patients approached, 24 were recruited, while all caregivers (n=21) and clinicians (n=34) approached were recruited. A total of 144 specialist visits were recorded, approximately 12 specialist visits at a median of one MDC visit per patient. Of the recorded patients, 75% (9/12) viewed videos. High median intervention feasibility (4, SD 0.99) and acceptability (4, SD 1.22) of intervention measures were reported by patients and caregivers in the intervention arm. High median intervention feasibility (5, SD 0.21) and acceptability (4.88, SD 0.4) were reported by clinicians. Of the 24 patients, 50% (n=12) did not complete a 3-month follow-up, primarily due to death (n=10).

**Conclusions:**

Video recording is highly feasible and acceptable for patients, caregivers, and clinicians at a rural ALS clinic. Our level of attrition is a useful benchmark for future studies in MDC populations. Despite high rates of patient death, 1-week assessments highlight the value of recordings for both patients and caregivers.

**Trial Registration:**

ClinicalTrials.gov NCT04719403; https://clinicaltrials.gov/study/NCT04719403

## Introduction

Amyotrophic lateral sclerosis (ALS) is a neurodegenerative disorder causing progressive loss of motor functioning, typically leading to death within 5 years of symptom onset [1-3]. Patients and their caregivers require information from multiple clinical specialties to guide treatment plans involving interrelated aspects of their disease [4]. Multidisciplinary clinics (MDCs) where patients meet with up to 12 specialists sequentially during a 3- to 4-hour visit are the gold-standard approach to care management [5]. Given the duration and complexity of MDC visits, less than 40% of treatment recommendations are recalled by patients [6,7]. This also impacts caregivers who report stress when they do not have enough information about managing the patient’s condition [8,9].

MDCs currently provide patients with brief, written after-visit summaries (AVSs) of their treatment plan from each specialist, yet the AVSs are not comprehensive and are typically written in ways that are not easily understood by patients [10,11]. Sharing video recordings of clinic visits is an innovative strategy that may assist with addressing this knowledge gap by providing a more detailed and accurate record than memory alone [12]. While not studied in ALS, access to visit recordings has been linked to better self-management and treatment adherence in other chronic conditions [13].

We conducted a randomized controlled pilot trial to determine the feasibility and acceptability of video recording in an ALS MDC setting. By understanding whether recordings are feasible or acceptable, the results of this pilot study will help determine whether these modalities have potential for future study. These findings will inform the development of future trials that are inclusive of patients with ALS and their caregivers, which may help facilitate recall and understanding of visit information.

## Methods

### Design

We conducted a randomized controlled pilot trial. Participants were allocated to receive their usual AVS or their usual AVS plus video recording of their ALS MDC visit (video) on a 1:1 ratio using variable block randomization with block sizes of 4 and 2. The randomization sequence was produced programmatically in R (version 3.4; R Core Team) by RWRB, and participants were randomly assigned upon completion of the baseline assessment.

### Setting

Participants were recruited from the Dartmouth Health Heater Road clinic. The MDC occurred each month, with clinicians rotating between rooms based on a preset clinician schedule with visit times approximating 30-40 minutes.

### Participants

#### Clinic Staff

Clinical staff were recruited in January 2021 during a pre-MDC team meeting; we aimed to recruit, consent, and survey all clinicians, though not all clinicians attended every MDC visit or met with each patient.

#### Patients and Caregivers

We included patients aged ≥18 years who communicated in English, were able to use a computer to access recordings, and were primarily treated for ALS. Caregivers, aged ≥18 years, were identified by patients as a family member or friend who assists with their health and health care. Patients were sent letters describing the research project 3 weeks before their visit and given the opportunity to opt out. Between 7 and 10 days before their visit, potential patients (and their caregivers) were contacted by telephone to determine their interest and eligibility. Eligible patients and caregivers could complete consent in-person or remotely. Post consent, patients completed a baseline assessment and were then randomly allocated to the treatment as usual (TAU) arm or video arm.

### TAU Arm

In the TAU arm, patients and their caregivers met with clinicians as normal, with no change to clinic procedures.

### Audio or Video Intervention

All MDC visits during the 3-month study period for each participant in the video arm were recorded on a Canon Vixia camcorder and RØDE microphone. Patients accessed their recordings through HealthPal. HealthPAL is an National Institutes of Health–funded, open-source, HIPAA (Health Insurance Portability and Accountability Act)–compliant clinic visit recording, storage, and sharing platform developed by a member of the research team, Barr et al [6] at Dartmouth College [14]. The intervention underwent no changes during the trial. The camera was set up to capture the clinical encounter in-frame, including the patient, caregiver, and clinician or clinicians. A secure, password-protected HealthPAL account was created for each patient that could be accessed within 48 hours of the clinic visit. In HealthPAL, each clinician visit was labeled by clinician, clinician specialty, and date to allow easier navigation of the MDC recording. Though not required in our protocol, recordings could be shared with caregivers by the patient inviting the caregiver to create their own account or by watching the videos on the patient’s account.

### Study Procedures

At the clinic, a research associate managed the recording procedure, including setting up and turning on and off the recording device. Within 2 days of the visit, patients would receive an email directing them to HealthPAL to access their recordings. Participants completed web-based follow-up surveys and interviews at 1-week and 3-month interval to explore factors related to the feasibility of our research protocol and the acceptability of recording. Participating patients received an honorarium of US $20 for completing each assessment and US $30 for completing the poststudy qualitative interview.

### Patient and Caregiver Interviews

Patients and caregivers in the video arm were invited to complete semistructured interviews informed by the Consolidated Framework for Implementation Research to better understand how they used the video recordings, barriers and facilitators to recording implementation, and the impact of the recording on clinic visit interactions (Multimedia Appendix 1) [15]. Participants were given the option to receive an email containing questions from the interview guide; this option was implemented in response to participant functional limitations, as ALS disease progression precludes many patients from speaking effectively [16]. Follow-up surveys and interviews were completed by RWRB, a male researcher with qualitative methods training, and transcripts were audio-transcribed using TranscribeMe.

### Clinician Surveys

At the conclusion of the project, a 6-question follow-up survey was distributed to all regular clinic staff at preclinic meetings to assess the feasibility, acceptability, and appropriateness of video and open-ended questions to identify challenges associated with recording and their potential effects on practice style and patient encounters [17].

### Outcomes

The primary outcome measures were the feasibility and acceptability of recording. We used 2 validated surveys, the Feasibility of Intervention Measure and Acceptability of Intervention Measure [17]. We prespecified a target median Feasibility of Intervention Measure and Acceptability of Intervention Measure of ≥3 out of 5 based on published thresholds to indicate feasibility or acceptability [17]. We also assessed acceptability and feasibility based on the actual review of the video recordings. Based on previous guidelines, we determined that a 70% or higher viewing rate would indicate high feasibility and acceptability [18]. We collected preliminary data on patient and caregiver behavioral and health-related outcomes, including anxiety, depression, and adherence to exercise and medications, using validated patient-reported surveys.

### Analysis

We aimed to recruit a sample of 24 patients and at least 12 caregivers, as 10-30 participants is adequate to detect feasibility issues and gather preliminary quantitative outcome data [19].

### Quantitative Analysis

In the process of determining feasibility and acceptability, we collected exploratory outcome data to determine whether participants would complete baseline and follow-up surveys. We present the results of tests of differences using the Mann-Whitney *U* test between the intervention and TAU at each timepoint and the results of a repeated-measures ANOVA between baseline and 1 week. Repeated measures were not conducted at the 3-month timepoint due to attrition. All analyses and the randomization sequence were conducted using R.

### Qualitative Analysis

RWRB performed directed qualitative content analysis of the interview transcripts using a codebook consisting of a priori domains, including attitudes and behaviors related to recording use, sharing recordings, and the impact of recordings on clinic visits [20]. Transcripts were reviewed and coded by RWRB. Coded transcripts were audited by LM to assess alignment of text excerpts with the attached code. Disagreements were resolved by PJB. Text excerpts were aggregated by code; code reports were reviewed for prominent and salient patterns to develop themes. Field notes from RWRB were also analyzed in the qualitative analysis.

### Ethical Considerations

This study received ethical approval from the Dartmouth Health Institutional Review Board (02000798) and was registered on ClinicalTrials.gov (NCT04719403) in January 2021. Recruitment began in April 2021 and continued through March 2022. Participants were consented to according to Dartmouth Health protocols and were given paper and electronic copies of consent forms for review. Data collection concluded in June 2022. Deidentified data are stored on protected and secured hard drives at Dartmouth College. This study adhered to the CONSORT (Consolidated Standards of Reporting Trials) extension for pilot trials (Multimedia Appendix 2) [15].

## Results

### Overview

All clinicians at the MDC agreed to take part, including the regularly scheduled staff (n=14) as well as rotating residents, fellows, and ad hoc specialists (total clinicians n=34). We approached 30 patients to meet our targeted sample of 24 participants (80% recruitment rate; 22 through e-consent and 2 through in-person consent; CONSORT flow diagram, Multimedia Appendix 2). In TAU, demographic information for 2 patients was unavailable: one patient entered hospice care before their first visit, while another did not receive the demographic questionnaire. About 65% (14/22) were male patients with a median age of 66.5 years (Table 1). Of the 12 participants in the intervention arm, study assessments were completed by 11 at 1 week and 7 participants at 3 months. Of the 12 participants in TAU, study assessments were completed by 9 at 1 week and by 5 participants at 3 months. More information is given in Multimedia Appendix 2. Attrition was primarily due to death (n=6 in TAU and n=4 in the intervention arm), while one patient was dissatisfied with their allocation to TAU and one newly diagnosed patient withdrew to focus on managing their condition. We recruited 100% of caregivers approached (n=21 caregivers), with 12 in the intervention arm and 9 in TAU. About 70% (14/21) were female caregivers, with a median age of 58.5 years (Table 1). After patients passed away, caregivers were not contacted further.

**Table 1:** Patient and caregiver characteristics by intervention.

Characteristics		Patients				Caregivers		
		Video (n=12)	TAU^a^ (n=11)	Total (n=22)	Video (n=12)		TAU (n=9)	Total (n=21)
**Age (years)**								
	Mean (SD)	63.67 (14.71)	64.8 (9.93)	64.182 (12.489)	64.2 (10.5)		55 (5)	60.1 (9.5)
	Range	30-81	52-79	30-81	46-80		46-63	46-80
**Gender, n (%)**								
	Women	4 (33)	4 (40)	8 (36)	7 (64)		7 (78)	14 (70)
	Men	8 (67)	6 (60)	14 (64)	4 (36)		2 (22)	6 (30)
**Income (US $), n (%)**								
	<50,000	3 (27)	4 (40)	7 (33)	2 (18)		3 (33)	5 (25)
	50,000-99,999	5 (46)	4 (40)	9 (43)	6 (55)		2 (22)	8 (40)
	>100,000	3 (27)	2 (20)	5 (24)	3 (27)		4 (44)	7 (35)
**Education, n (%)**								
	Less than college	3 (25)	4 (40)	7 (32)	6 (55)		5 (56)	11 (55)
	College degree	4 (33)	3 (30)	7 (32)	3 (27)		0 (0)	3 (15)
	More than college	5 (42)	3 (30)	8 (36)	2 (18)		4 (44)	6 (30)
**Literacy level, n (%)**								
	Not high	6 (50)	6 (60)	12 (56)	2 (18)		1 (13)	3 (14)
	High	6 (50)	4 (40)	10 (46)	9 (82)		7 (88)	16 (84)

^a^TAU: treatment as usual.

### Visit Recordings

A total of 144 unique encounters with clinicians were recorded, for approximately 12 recordings per patient in the intervention arm. Of patients receiving recordings, 75% (9/12) viewed at least one video, with an average of 6 videos viewed each. One participant did not think they required videos, one patient did not want to watch the videos, and one patient’s condition progressed quickly, and they did not feel the videos were relevant. Of the 55 videos watched, 36% (n=20) were in neurology, and 29% (n=16) were in physical and occupational therapy. Of note, 3 caregivers continued watching videos after patients had passed away.

### Quantitative Results

Mean intervention feasibility (4, range 2-5, 95% CI 3.05-4.7) and acceptability (4, range 2-5, 95% CI 2.79-4.83) of intervention measures were reported by patients in the intervention arm. While not statistically significant, the video arm performed better in adherence to refills and medications, adherence to exercise, caregiver burden, and caregiver preparedness at 1 week compared to TAU (Table 2). All 14 regularly scheduled clinicians at the MDC responded to the survey. Mean intervention feasibility (5, range 3-5) and acceptability (4.5, range 3-5) were reported at the conclusion of the trial.

**Table 2:** Study outcome measures by intervention arm at baseline, 1 week, and 3 months (t tests).

Measures				Baseline		1-week		3-months
**Patient outcomes**								
	**Patient Satisfaction Questionnaire-18 [21]**							
		Video, mean	7.75		7.64		8.29	
		Control, mean	6.73		6.7		5.7	
		*P* value	.19		.29		.23	
		95% CIs	–0.58 to 2.62		–0.92 to 2.86		–2.83 to 7.75	
	**Adherence to refills and medications-7 [22]**							
		Video, mean	24.67		25.27		25.67	
		Control, mean	24.55		24.3		25	
		*P* value	.86		.06		.48	
		95% CIs	–1.32 to 1.56		–0.02 to 1.90		–3.96 to 2.37	
	**Exercise Adherence Rating Scale [23]**							
		Video, mean	10		11.82		10.42	
		Control, mean	10.27		10.22		10	
		*P* value	.80		.11		.56	
		95% CIs	–2.55 to 2.01		–0.42 to 3.61		–4.13 to 6.38	
	**General Anxiety Disorder-7 [24]**							
		Video, mean	4		—^a^		4.28	
		Control, mean	5		—		2	
		*P* value	.61		—		.33	
		95% CIs	–5.03 to 3.03		—		–2.3 to 5.8	
	**Patient Health Questionnaire-8 [25]**							
		Video, mean	6		—		6.375	
		Control, mean	5.8		—		3	
		*P* value	.92		—		.19	
		95% CIs	–3.62 to 3.98		—		–2.06 to 8.81	
	**Caregiver outcomes**							
	**Burden Scale for Family Caregivers [26]**							
		Video, mean	18.33		18.81		21.67	
		Control, mean	24.87		25.14		24.25	
		*P* value	.04		.01		.48	
		95% CIs	–12.71 to –0.38		–10.69 to –1.96		–10.7 to –5.54	
	**Preparedness for Caregiving Scale [27]**							
		Video, mean	18.33		19.27		18.5	
		Control, mean	17.5		17.85		20.25	
		*P* value	.77		.7		.69	
		95% CIs	–5.08 to 6.75		–6.33 to 9.17		–11.85 to 8.34	
	**Caregiver General Anxiety Disorder-7 [24]**							
		Video, mean	4.58		4.36		4	
		Control, mean	11.13		6.43		7	
		*P* value	.04		.41		.41	
		95% CIs	–12.55 to –0.53		–7.39 to 3.26		–12.31 to 6.31	
	**Caregiver Patient Health Questionnaire-8 [25]**							
		Video, mean	3.41		3.81		4.83	
		Control, mean	9.63		6.71		4.75	
		*P* value	.02		.17		.98	
		95% CIs	–11.11 to –1.3		–7.29 to 1.49		–6.59 to 6.75	

^a^Not applicable.

### Qualitative Results

We received responses from 6 patient-caregiver dyads after reaching out to 8 dyads for interest in completing an interview, resulting in 2 interviews lasting approximately 20 minutes and 4 written responses. All 14 regularly scheduled clinicians completed the clinician survey. Clinicians did not report detrimental effects of visit recordings or study procedures on patient visits. Some clinicians perceived a greater sense of accountability and awareness of their actions during the recorded visits, though clinicians thought this was beneficial and should be replicated in their nonrecorded visits. Clinicians also expressed a desire to view the recordings for their own clinical improvements.

The qualitative analysis yielded 3 primary themes in relation to recording use, sharing recordings, and the impact of recording on clinic visits. Refer to Table 3 for full qualitative results and illustrative quotes.

**Table 3:** Qualitative analytic domains, quotes numbers, and quotes.

Domain and quote number		Quote
**Recording use**		
	1	You get old and you forget, but you have the ability to go back to the video and say, “Oh, that’s what (the doctor) meant.” (Patient)
	2	Watching, I was like “I don’t remember them saying that. Boy I’m glad I watched because I didn’t write it down when I take notes, and I didn’t remember it.” (Caregiver)
	3	Oh my goodness, I didn’t focus on that while I was there...Person number 1 was talking and person number 2 had an interjection that was helpful, but I didn’t pick up on it. So the video actually brought that to light, which was fantastic. (Caregiver)
	4	To remind myself how the appointments went so that I could formulate some questions I might ask at the second appointment. It kind of refreshed my mind about what we had talked about and what questions I had now that may have changed over three months. (Patient)
**Sharing recordings**		
	5	The drawback (of recordings) for me was seeing myself and noticed (sic) I had declined! L Pertaining to my looks, and speech problems! That was not a bad thing, because I already knew the decline! It was important to me to know and observe the progress of my ALS! (Patient)
	6	I have a tendency to just kind of go through and deny stuff or not always say when I was comfortable. I was pretty honest at the clinic visits, so (my caregiver) gets to hear the honest me communicating as well as, “Oh, hey, have you been doing those exercises they told you to do in PT?” (Patient)
	7	I have a friend who was very interested in going to some clinic visits with me...I was able to tell her, “No, you don’t need to spend four hours of your time coming to clinic. If you want to, we can review these video tapes.” (Patient)
	8	But as time goes on, it might be really helpful for some of my good friends to understand more about what’s happening with me. They’re very important members of my care team. (Patient)
**Impact on clinic visits**		
	9	Didn’t have any impact on my care. (Patient)
	10	Quick and Easy. Low impact on doctor visits. (Caregiver)
	11	Oh, I don’t think (recording) - I don’t think (recording) affected (the visit) at all. I think we were tuned in to each person that came in to talk to (us), and we focused on what was being said. I don’t think either of us paid much attention to the fact you’re recording it at all...It didn’t take away from the visit. (Caregiver)
	12	It’s hard for me to write...and taking notes kind of distracts me while I’m interacting. So it was very helpful for me not to have to take notes once I realized that I’d be able to just look at the video tapes later on. (Patient)

### Recording Use

Participants used recordings to augment clinical encounters and monitor their condition. One patient said, “You get old, and you forget, but you have the ability to go back to the video and say, ‘Oh, that’s what (the doctor) meant.”

Visits often occurred every 3 months, and participants found it useful to revisit material when planning for their next visit.

To remind myself how the appointments went so that I could formulate some questions I might ask at the second appointment. It kind of refreshed my mind about what we had talked about and what questions I had now that may have changed over three months.

Patient

Participants also used the recordings to assess the progression of their condition. They found it useful to see themselves rather than just rely on how they were feeling. This was echoed by another participant, who said that the video “kept them honest” when communicating about their condition with their caregivers and physical therapists.

I have a tendency to just kind of go through and deny stuff or not always say when I was comfortable. I was pretty honest at the clinic visits, so (my caregiver) gets to hear the honest me communicating as well as, ‘Oh, hey, have you been doing those exercises they told you to do in PT?’

Patient

### Sharing Recordings

Sharing recordings supported patient independence and facilitated updates to family and friends. Recordings were shared to engage family members and friends who could not attend visits. They were used to keep family members informed and engaged with the visits; indeed, one patient was scheduled off the waitlist and their caregiver could not attend, but the patient chose to attend the visit alone, with the knowledge that their caregiver would receive the information they needed from the videos; without the video, the patient would not have elected to attend the clinic.

I have a friend who was very interested in going to some clinic visits with me...I was able to tell her, ‘No, you don’t need to spend four hours of your time coming to clinic. If you want to, we can review these video tapes.’

Patient

### Impact of Recording on Clinic Visits

Clinical workflow and interactions were not reported to be impacted by recording. All participants mentioned they found no detrimental impact of video recordings on clinic visit interactions. Participants mentioned the positive effects of recording, with one participant mentioning that the ability to watch the recording instead of taking notes allowed them to be less distracted during the visit and focus on interacting with clinicians.

Oh, I don’t think (recording)-I don’t think (recording) affected (the visit) at all. I think we were tuned in to each person that came in to talk to (us), and we focused on what was being said. I don’t think either of us paid much attention to the fact you’re recording it at all...It didn’t take away from the visit.

Caregiver

## Discussion

### Principal Findings

In our sample, patients, caregivers, and clinicians found video recording to be highly acceptable and feasible to implement at ALS MDCs. While we experienced attrition, we found recordings were used by patients and their caregivers to remember what was said in visits and to share information from visits with family members.

### Limitations

While we experienced high attrition, this was an acceptable risk when designed the pilot project. Patients with advanced ALS are often excluded from research [28], often to protect researchers from attrition, despite the potential for interventions to provide benefit. In this case, we decided video recordings, which focus on communication, could be valuable to patients at any stage of disease. While we did not capture time since diagnosis, this information could be an important moderator to future use of clinic visit recordings. Additionally, our pilot trial adds an important contribution to the field by identifying the level of attrition one can expect when including patients with advanced ALS in order to inform sample size estimates for future efficacy trials. The high attrition rate of our participants, particularly at 3-month follow-up, is a limitation for our quantitative analysis. However, this study is a pilot that seeks to understand how to design a larger trial and addresses a gap in current ALS research where patients with more advanced disease are often excluded from trials [28]. This trial occurred at a single site at a rural MDC; future trials should include clinics in urban environments to determine whether our positive findings may be generalizable. Despite the rural setting of the MDC, none of our patients or caregivers reported problems accessing or viewing the videos, indicating they had reasonably strong broadband internet connections. Further work is needed to determine potential solutions to sharing video recordings of clinic visits with those who may not have robust internet connections.

We chose not to mask at this stage as the trial was pilot based in nature and not focused on efficacy. Because patients all chose to watch videos with caregivers, we do not know whether patients or caregivers initiated the viewing or what proportion of video views were initiated by patients or caregivers. While we received 6 responses to 8 requests for qualitative interviews, it is unknown whether the experiences of the 2 who did not respond may have differed from those who did.

### Comparison With Previous Work

Providing video recordings to patients is a promising method of providing information from clinic visits to patients and caregivers that has been well received in disciplines such as oncology, pediatrics, cardiac surgery, and orthopedic surgery [29]. Our results are similar to those from other fields, indicating that video recording is highly feasible and acceptable for patients with ALS and their caregivers at MDCs. In our sample, video recordings helped overcome the limitations of other methods of communicating visit information by offering a record of what was said without patients or caregivers needing to rely on imperfect recall or notes, and they showed the clinician’s best attempt to communicate the information rather than written medical jargon [30,31].

Adherence to exercise and medication is important in the management of chronic disease and is connected to improved outcomes in patients with ALS [2,32]. The pilot was not designed to detect statistically significant differences, though results were promising for both adherence to medications and adherence to exercise in the video arm—similar to the use of recordings in other fields [33]. This is supported by the video usage statistics, where neurology visits (medications are discussed) and physical and occupational therapy visits (exercises are discussed) were the most-watched specialties. Our qualitative findings also highlight the potential value of video for exercise adherence in patients with ALS and their caregivers, with patients and caregivers noting to the research assistant the utility of seeing the exercise demonstrated on camera.

Despite the benefits of ALS MDCs, the length of visits and amount of information exchanged have been described as burdensome [34]. Our findings indicate that video recordings could make these visits easier. Both patients and caregivers found that having a recording lessened the note-taking burden because the recording functioned as a perfect record of the visit. By reducing “patient work” in visits, patients and caregivers could be more engaged with their clinicians during visits when they have videos to watch later; further work could understand the impact of recordings on communication in clinic visits. The visit may also be of higher quality—clinicians reported a greater sense of accountability, supporting findings from a systematic review of visit recording [35].

Caregivers of patients with ALS have reported strain associated with feeling less supported by the care team [8]. A scoping review of 33 recording studies found that while patients often shared recordings with caregivers, caregivers were rarely enrolled in the studies, and thus their experience with recording and its impact on outcomes are not well documented [29]. We found caregivers were enthusiastic about the recordings and used them to assist their caregiving, which could be supporting the pathway where a better understanding of treatment and diagnosis leads to improved caregiver performance [36].

Future work could explore the impacts of recording on caregiver outcomes identified in the scoping review, including resilience and coping. The utility of clinic visit recordings as mementos or their effects on bereavement is similarly less understood; videos have been identified as valuable by caregivers after patients have passed away, but the identified videos included home movies and personal videos rather than those occurring in a medical encounter [37].

### Implications

With significant developments in recording technology and usage practices, video-recorded clinic data can assist patient and caregiver management and, by extension, improve health outcomes. While other methods of visit communication may be inadequate for patients with ALS and their caregivers because of low visit recall and jargon-heavy summaries [38], video recordings provide a feasible alternative, especially when taken in conjunction with natural language processing algorithms to facilitate understanding. These results lay a foundation that could change the way information is communicated for patients with ALS and their caregivers at the 73 American ALS MDCs. Video recordings could help address issues associated with long or strenuous visits by reducing the burden of documentation on patients, caregivers, and clinicians.

### Conclusions

Our results indicate that recording interventions is feasible and acceptable in our sample of patients, caregivers, and clinicians at a multidisciplinary ALS clinic. A larger-scale trial with flexible follow-up assessment is required to further explore the clinical utility of recordings. As recording technology is refined, recordings could have great utility for patients, their caregivers, and the clinical team.

## Appendix

Multimedia Appendix 1 Interview guide.

Multimedia Appendix 2 Participant flow diagram.

Multimedia Appendix 3 CONSORT-eHEALTH checklist (V 1.6.1).

## Abbreviations

ALS: amyotrophic lateral sclerosis
AVS: after-visit summary
CONSORT: Consolidated Standards of Reporting Trials
HIPAA: Health Insurance Portability and Accountability Act
MDC: multidisciplinary clinic
TAU: treatment as usual

## Data Availability

The data sets and analyses generated during the study are available from the corresponding author upon reasonable request.
