# Supplementary material for: The Feasibility and Acceptability of Sharing Video Recordings of Amyotrophic Lateral Sclerosis Clinical Encounters With Patients and Their Caregivers: Pilot Randomized Clinical Trial
Source: JMIR Form Res. 2024 Jun 26;8:e57519. doi: 10.2196/57519 (PMC11237769; doi:10.2196/57519)
Supplement: Multimedia Appendix 1 [file formative_v8i1e57519_app1.pdf]

Determining the feasibility, acceptability, and potential effectiveness of sharing video recordings of multidisciplinary ALS clinic visits

Qualitative Interview Guide - Dyad

Introduction

For the last three months, [patient name] has been in a study in which you were both given access to a video recording of your clinic visits. I would like to talk with you both today about your experiences in the study, including what worked well, barriers or challenges you may have experienced, and how recording and listening to your clinic visits impacted your clinical care, your health, and your caregiving. I am interested in understanding these things from *your point of view*, from *your perspective*.

What we talk about during this interview is confidential. I will not discuss what you share during this interview with anyone except other members of the research team. I ask that you please try to be as honest and open as you can so we can learn from your experience. It's important that we learn from experiences that went well and also things that may have been challenging.

If there are questions that you do not feel comfortable answering or discussing, you do not have to answer them. Please tell me and we'll move on to the next question. If you need or want to take a break at any time, please let me know. If you get tired and would like to continue the interview at another time, please let me know.

This interview is happening with both [patient name] and [caregiver name]. For most questions, I'll address both of you; one person can take the lead in answering each question, and the other can feel free to elaborate on each other's responses and jump in when you have something to say. For some questions, I will ask specifically for either [patient name] or [caregiver name] to respond, but please feel free to elaborate on those answers.

This interview will take about 20 minutes of your time. Before we go on, do you have any questions for me?

Experience in the Study

As you know, for the past month, [patient name] has been in a study in which we recorded your clinic visits. I'd like to start the interview by asking you to describe your experience in the study.

1. First, please tell me in your own words, about your experience in the study. [Invite patient to speak in an open-ended way for 1-2 minutes, then ask caregiver to respond. Interviewer is listening for overall impressions, emotional valence, challenges to help guide probing throughout interview.]

2. Thank you so much. When you first heard about the study, what were your initial impressions?

3. What made you decide to participate in the study?
- Did you talk with your doctor or nurse about it?
- Did you talk with family members or friends?
- How did these conversations influence your decision to take part?

Before you were in the study, you met with a Research Assistant who explained the purpose of the research and what you would be asked to do to participate.

4. What worked well about that first meeting?

5. What was challenging about that meeting?

- Was the conversation with the Research Assistant clear and understandable or was it confusing to you?

6. Do you have suggestions for how we could improve the first meeting with the Research Assistant to ensure that people understand the study?

During clinic visits, the Research Assistant was in the exam room to record the visits.

10. What worked well about that process?

11. What was challenging or inconvenient about that process?

12. Do you have any suggestions for how this process could be improved?

Following your appointment, you were given access video recording of the clinic visit, and instructions on how to use the recording system.

13. How easy or hard was it to follow those instructions?
- What worked well about that process?
- What was challenging about that process?

14. What would have helped you in this process? Do you have any suggestions for how this process could be improved?

<u>Experience with the Intervention</u>

We’ve talked some about your experience with recording your clinic visits. Now I’d like to learn more about your perspectives on having the recording of clinic visits and how

this may have impacted your clinical care, how you manage your illness, and the caregiving process.

1. First, please tell me in your own words about your experience of having a recording of your clinic visit. [Invite participant to speak in an open-ended way for 1-2 minutes.]

2. How did you use the recording? Under what circumstances did you use the recording?

    - Probe for self/caregiver/clinician/study- initiated use.

    - Could you give me an example of a time that you used the recording?

    -What type of device did you use to watch the recording?

    - What challenges, if any, did you have in using the recording?

    -Did you listen together/separately? Why?

    -Were there any challenges to listening together?

    If multiple visits were recorded

    -Did you watch the recording prior to the second visit? Why or why not?

3. Did you share your recordings with anyone else? With whom? Under what circumstances?

4. From your perspective, what benefits, if any, were there to recording the clinic visits?

5. From your perspective, what drawbacks, if any, were there to recording the clinic visits?

6. How did recording your clinic visits impact your clinical care?

    - How did recording impact communication with your clinicians?

    - What changes, if any, did you notice in your clinical care?

7. How did recording your clinic visit impact how you manage your illness?

    - What, if anything, did you do differently to manage your illness since having recordings of your clinic visits?

    - What changes in your health, if any, have you noticed?

    PT Question

    -Did you notice a difference in care when you have the recording?

    CG Question

-Did having access to the recordings affect your caregiving?

8. Would you recommend recording clinical visits to others? Why/why not?

<u>Closing</u>

I want to thank you both for sharing your experiences with me.

Before we stop for today, are there any things that you'd like me to know about your experiences in the study that we haven't covered?

Thank you so much for your time today.
