# Supplementary material for: The Feasibility and Acceptability of Sharing Video Recordings of Amyotrophic Lateral Sclerosis Clinical Encounters With Patients and Their Caregivers: Pilot Randomized Clinical Trial
Source: JMIR Form Res. 2024 Jun 26;8:e57519. doi: 10.2196/57519 (PMC11237769; doi:10.2196/57519)
Supplement: Multimedia Appendix 2 [file formative_v8i1e57519_app2.docx]

Assessed for eligibility (n=30)

Excluded (n=6)

Declined to participate (n=6)

Assessed for Time 1 (n=11)

Assessed for Time 2 (n=8)

Etc ..

Lost to follow-up (give reasons) (n=3)

3 participants died before completion

Discontinued intervention (give reasons) (n=1)

1 participant declined to continue participating after their prognosis

Allocated to intervention (n=12)

 Received allocated intervention (n=12)

Lost to follow-up (give reasons) (n=6)

6 participants died before completion

Discontinued usual care (give reasons) (n=1)

1 participant wanted the video arm

Allocated to usual care (n=12)

Received usual care (n=12)

Assessed for Time 1 (n=9)

Assessed for Time 2 (n=5)

## Allocation

## Assessment

## Follow-Up

Randomized (n=24)

## Enrollment

Screened before eligibility assessment (n=30)

Excluded (n=0)

## Screened
