# Supplementary material for: The Feasibility and Acceptability of Sharing Video Recordings of Amyotrophic Lateral Sclerosis Clinical Encounters With Patients and Their Caregivers: Pilot Randomized Clinical Trial
Source: JMIR Form Res. 2024 Jun 26;8:e57519. doi: 10.2196/57519 (PMC11237769; doi:10.2196/57519)
Supplement: Multimedia Appendix 3 [file formative_v8i1e57519_app3.pdf]

CONSORT-EHEALTH Checklist V1.6.2 Report	Manuscript Number	57519
**(based on CONSORT-EHEALTH V1.6), available at [http://tinyurl.com/consort-ehealth-v1-6].**		
**Date completed**		
6/25/2024 12:40:19		
**by**		
Reed Bratches		
The Feasibility and Acceptability of Sharing Video Recordings of Amyotrophic Lateral Sclerosis Clinical Encounters with Patients and their Caregivers: Pilot Randomized Clinical Trial		
TITLE		
**1a-i) Identify the mode of delivery in the title**		
"The Feasibility and Acceptability of Sharing Video Recordings of Amyotrophic Lateral Sclerosis Clinical Encounters With Patients and Their Caregivers: Pilot Randomized Clinical Trial"		
**1a-ii) Non-web-based components or important co-interventions in title**		
"we did not have non-web based intervention components"		
**1a-iii) Primary condition or target group in the title**		
"The Feasibility and Acceptability of Sharing Video Recordings of Amyotrophic Lateral Sclerosis Clinical Encounters With Patients and Their Caregivers: Pilot Randomized Clinical Trial"		
ABSTRACT		
**1b-i) Key features/functionalities/components of the intervention and comparator in the METHODS section of the ABSTRACT**		
"Patients and their caregivers were randomized to either receive their normal after-visit summary (treatment as usual) or to receive their normal after-visit summary and a video recording of their MDC visit (video). Each specialist visit had its own recording and was accessible by patients and caregivers using a secure web-based platform called HealthPAL over a 3-month follow-up period."		
**1b-ii) Level of human involvement in the METHODS section of the ABSTRACT**		
"Patients and their caregivers were randomized to either receive their normal after-visit summary (treatment as usual) or to receive their normal after-visit summary and a video recording of their MDC visit (video). Each specialist visit had its own recording and was accessible by patients and caregivers using a secure web-based platform called HealthPAL over a 3-month follow-up period. "		
**1b-iii) Open vs. closed, web-based (self-assessment) vs. face-to-face assessments in the METHODS section of the ABSTRACT**		
"This study was a randomized, controlled pilot trial with 3 months of follow-up from April 2021 to March 2022 in a rural multidisciplinary neurology clinic. We recruited patients with ALS, their caregivers, and their clinicians."		
**1b-iv) RESULTS section in abstract must contain use data**		
"Of the recorded patients, 75% (9/12) viewed videos. High median intervention feasibility (4, SD 0.99) and acceptability (4, SD 1.22) of intervention measures were reported by patients and caregivers in the intervention arm. High median intervention feasibility (5, SD 0.21) and acceptability (4.88, SD 0.4) were reported by clinicians. Of the 24 patients, 50% (n=12) did not complete a 3-month follow-up, primarily due to death (n=10)."		
**1b-v) CONCLUSIONS/DISCUSSION in abstract for negative trials**		
"This was not a negative trial"		
INTRODUCTION		
**2a-i) Problem and the type of system/solution**		
"MDCs currently provide patients with brief, written after-visit summaries (AVSs) of their treatment plan from each specialist, yet the AVSs are not comprehensive and are typically written in ways that are not easily understood by patients [10,11]. Sharing video recordings of clinic visits is an innovative strategy that may assist with addressing this knowledge gap by providing a more detailed and accurate record than memory alone [12]. While not studied in ALS, access to visit recordings has been linked to better self-management and treatment adherence in other chronic conditions [13]."		
**2a-ii) Scientific background, rationale: What is known about the (type of) system**		
"While not studied in ALS, access to visit recordings has been linked to better self-management and treatment adherence in other chronic conditions [13]."		
**Does your paper address CONSORT subitem 2b?**		
"We conducted a randomized controlled pilot trial to determine the feasibility and acceptability of video recording in an ALS MDC setting. By understanding whether recordings are feasible or acceptable, the results of this pilot study will help determine whether these modalities have potential for future study."		
METHODS		
**3a) CONSORT: Description of trial design (such as parallel, factorial) including allocation ratio**		
"We conducted a randomized controlled pilot trial. Participants were allocated to receive their usual AVS or their usual AVS plus video recording of their ALS MDC visit (video) on a 1:1 ratio using variable block randomization with block sizes of 4 and 2. "		
**3b) CONSORT: Important changes to methods after trial commencement (such as eligibility criteria), with reasons**		
"We did not change criteria after the trial began"		
**3b-i) Bug fixes, Downtimes, Content Changes**		
"There were no staff changes or system failures/downtime"		
**4a) CONSORT: Eligibility criteria for participants**		
"We included patients aged ≥18 years who communicated in English and were primarily treated for ALS. Caregivers, aged ≥18 years, were identified by patients as a family member or friend who assists with their health and health care. "		
**4a-i) Computer / Internet literacy**		
“We included patients aged ≥18 years who communicated in English, were able to use a computer to access recordings, and were primarily treated for ALS.”		
**4a-ii) Open vs. closed, web-based vs. face-to-face assessments:**		
". Patients were sent letters describing the research project 3 weeks before their visit and given the opportunity to opt out. Between 7 and 10 days before their visit, potential patients (and their caregivers) were contacted by telephone to determine their interest and eligibility. Eligible patients and caregivers could complete consent in-person or remotely. "		
**4a-iii) Information giving during recruitment**		
." Patients were sent letters describing the research project 3 weeks before their visit and given the opportunity to opt out. Between 7 and 10 days before their visit, potential patients (and their caregivers) were contacted by telephone to determine their interest and eligibility. Eligible patients and caregivers could complete consent in-person or remotely. "		
**4b) CONSORT: Settings and locations where the data were collected**		
"Participants were recruited from the Dartmouth Health Heater Road clinic. The MDC occurred each month, with clinicians rotating between rooms based on a preset clinician schedule with visit times approximating 30-40 minutes."		
**4b-i) Report if outcomes were (self-)assessed through online questionnaires**		
"Participants completed web-based follow-up surveys and interviews at 1-week and 3-month interval to explore factors related to the feasibility of our research protocol and the acceptability of recording."		
**4b-ii) Report how institutional affiliations are displayed**		
"participants were patients at the clinic; we did not brand the surveys or items."		
**5) CONSORT: Describe the interventions for each group with sufficient details to allow replication, including how and when they were actually administered**		
**5-i) Mention names, credential, affiliations of the developers, sponsors, and owners**		
"HealthPAL is an National Institutes of Health–funded, open-source, HIPAA (Health Insurance Portability and Accountability Act)–compliant clinic visit recording, storage, and sharing platform developed by a member of the research team, Barr et al [6] at Dartmouth College [14]"		
**5-ii) Describe the history/development process**		
"HealthPAL is an National Institutes of Health–funded, open-source, HIPAA (Health Insurance Portability and Accountability Act)–compliant clinic visit recording, storage, and sharing platform developed by a member of the research team, Barr et al [6] at Dartmouth College [14]"		
**5-iii) Revisions and updating**		

"HealthPAL is an National Institutes of Health–funded, open-source, HIPAA (Health Insurance Portability and Accountability Act)–compliant clinic visit recording, storage, and sharing platform developed by a member of the research team, Barr et al [6] at Dartmouth College [14]. The intervention underwent no changes during the trial.”		
**5-iv) Quality assurance methods**		
"At the clinic, a research associate managed the recording procedure, including setting up and turning on and off the recording device. Within 2 days of the visit, patients would receive an email directing them to HealthPAL to access their recordings. "		
**5-v) Ensure replicability by publishing the source code, and/or providing screenshots/screen-capture video, and/or providing flowcharts of the algorithms used**		
"HealthPAL is an National Institutes of Health–funded, open-source, HIPAA (Health Insurance Portability and Accountability Act)–compliant clinic visit recording, storage, and sharing platform developed by a member of the research team, Barr et al [6] at Dartmouth College [14]"		
**5-vi) Digital preservation**		
"we are unable to provide a link to the trial intervention though, as mentioned, the code is open-source."		
**5-vii) Access**		
"At the clinic, a research associate managed the recording procedure, including setting up and turning on and off the recording device. Within 2 days of the visit, patients would receive an email directing them to HealthPAL to access their recordings. Participants completed web-based follow-up surveys and interviews at 1-week and 3-month interval to explore factors related to the feasibility of our research protocol and the acceptability of recording. "		
**5-viii) Mode of delivery, features/functionalities/components of the intervention and comparator, and the theoretical framework**		
"the intervention has been described elsewhere; we include the citation to the intervention that includes these components."		
**5-ix) Describe use parameters**		
"At the clinic, a research associate managed the recording procedure, including setting up and turning on and off the recording device. Within 2 days of the visit, patients would receive an email directing them to HealthPAL to access their recordings. Participants completed web-based follow-up surveys and interviews at 1-week and 3-month interval to explore factors related to the feasibility of our research protocol and the acceptability of recording. "		
**5-x) Clarify the level of human involvement**		
"At the clinic, a research associate managed the recording procedure, including setting up and turning on and off the recording device. Within 2 days of the visit, patients would receive an email directing them to HealthPAL to access their recordings. Participants completed web-based follow-up surveys and interviews at 1-week and 3-month interval to explore factors related to the feasibility of our research protocol and the acceptability of recording. "		
**5-xi) Report any prompts/reminders used**		
"At the clinic, a research associate managed the recording procedure, including setting up and turning on and off the recording device. Within 2 days of the visit, patients would receive an email directing them to HealthPAL to access their recordings. Participants completed web-based follow-up surveys and interviews at 1-week and 3-month interval to explore factors related to the feasibility of our research protocol and the acceptability of recording. "		
**5-xii) Describe any co-interventions (incl. training/support)**		
"We had no co-interventions or trainings"		
**6a) CONSORT: Completely defined pre-specified primary and secondary outcome measures, including how and when they were assessed**		
"The primary outcome measures were the feasibility and acceptability of recording. We used 2 validated surveys, the Feasibility of Intervention Measure and Acceptability of Intervention Measure [17]. We prespecified a target median Feasibility of Intervention Measure and Acceptability of Intervention Measure of ≥3 out of 5 based on published thresholds to indicate feasibility or acceptability [17]. We also assessed acceptability and feasibility based on the actual review of the video recordings. Based on previous guidelines, we determined that a 70% or higher viewing rate would indicate high feasibility and acceptability [18]. "		
**6a-i) Online questionnaires: describe if they were validated for online use and apply CHERRIES items to describe how the questionnaires were designed/deployed**		
"The primary outcome measures were the feasibility and acceptability of recording. We used 2 validated surveys, the Feasibility of Intervention Measure and Acceptability of Intervention Measure [17]. We prespecified a target median Feasibility of Intervention Measure and Acceptability of Intervention Measure of ≥3 out of 5 based on published thresholds to indicate feasibility or acceptability [17]. We also assessed acceptability and feasibility based on the actual review of the video recordings. Based on previous guidelines, we determined that a 70% or higher viewing rate would indicate high feasibility and acceptability [18]. "		
**6a-ii) Describe whether and how “use” (including intensity of use/dosage) was defined/measured/monitored**		
"The primary outcome measures were the feasibility and acceptability of recording. We used 2 validated surveys, the Feasibility of Intervention Measure and Acceptability of Intervention Measure [17]. We prespecified a target median Feasibility of Intervention Measure and Acceptability of Intervention Measure of ≥3 out of 5 based on published thresholds to indicate feasibility or acceptability [17]. We also assessed acceptability and feasibility based on the actual review of the video recordings. Based on previous guidelines, we determined that a 70% or higher viewing rate would indicate high feasibility and acceptability [18]. "		
**6a-iii) Describe whether, how, and when qualitative feedback from participants was obtained**		
"Patients and caregivers in the video arm were invited to complete semistructured interviews informed by the Consolidated Framework for Implementation Research to better understand how they used the video recordings, barriers and facilitators to recording implementation, and the impact of the recording on clinic visit interactions (Multimedia Appendix 1) [15]. Participants were given the option to receive an email containing questions from the interview guide; this option was implemented in response to participant functional limitations, as ALS disease progression precludes many patients from speaking effectively [16]. Follow-up surveys and interviews were completed by RB, a male researcher with qualitative methods training, and transcripts were audio-transcribed using TranscribeMe."		
**6b) CONSORT: Any changes to trial outcomes after the trial commenced, with reasons**		
"Participants were recruited from the Dartmouth Health Heater Road clinic. The MDC occurred each month, with clinicians rotating between rooms based on a preset clinician schedule with visit times approximating 30-40 minutes."		
**7a) CONSORT: How sample size was determined**		
**7a-i) Describe whether and how expected attrition was taken into account when calculating the sample size**		
"Additionally, our pilot trial adds an important contribution to the field by identifying the level of attrition one can expect when including patients with advanced ALS in order to inform sample size estimates for future efficacy trials. "		
**7b) CONSORT: When applicable, explanation of any interim analyses and stopping guidelines**		
"The primary outcome measures were the feasibility and acceptability of recording. We used 2 validated surveys, the Feasibility of Intervention Measure and Acceptability of Intervention Measure [17]. We prespecified a target median Feasibility of Intervention Measure and Acceptability of Intervention Measure of ≥3 out of 5 based on published thresholds to indicate feasibility or acceptability [17]. We also assessed acceptability and feasibility based on the actual review of the video recordings. Based on previous guidelines, we determined that a 70% or higher viewing rate would indicate high feasibility and acceptability [18]. "		
**8a) CONSORT: Method used to generate the random allocation sequence**		
"The randomization sequence was produced programmatically in R (version 3.4; R Core Team) by RB, and participants were randomly assigned upon completion of the baseline assessment."		
**8b) CONSORT: Type of randomisation; details of any restriction (such as blocking and block size)**		
"The randomization sequence was produced programmatically in R (version 3.4; R Core Team) by RB, and participants were randomly assigned upon completion of the baseline assessment."		
**9) CONSORT: Mechanism used to implement the random allocation sequence (such as sequentially numbered containers), describing any steps taken to conceal the sequence until interventions were assigned**		
"The randomization sequence was produced programmatically in R (version 3.4; R Core Team) by RB, and participants were randomly assigned upon completion of the baseline assessment."		
**10) CONSORT: Who generated the random allocation sequence, who enrolled participants, and who assigned participants to interventions**		
"The randomization sequence was produced programmatically in R (version 3.4; R Core Team) by RB, and participants were randomly assigned upon completion of the baseline assessment."		
**11a) CONSORT: Blinding - If done, who was blinded after assignment to interventions (for example, participants, care providers, those assessing outcomes) and how**		
**11a-i) Specify who was blinded, and who wasn’t**		
Blinding was not possible for this trial		
**11a-ii) Discuss e.g., whether participants knew which intervention was the “intervention of interest” and which one was the “comparator”**		
"Eligible patients and caregivers could complete consent in-person or remotely. Post consent, patients completed a baseline assessment and were then randomly allocated to the treatment as usual (TAU) arm or video arm."		
**11b) CONSORT: If relevant, description of the similarity of interventions**		

Not relevant to our study		
**12a) CONSORT: Statistical methods used to compare groups for primary and secondary outcomes**		
"In the process of determining feasibility and acceptability, we collected exploratory outcome data to determine whether participants would complete baseline and follow-up surveys. We present the results of tests of differences using the Mann-Whitney U test between the intervention and TAU at each timepoint and the results of a repeated-measures ANOVA between baseline and 1 week. Repeated measures were not conducted at the 3-month timepoint due to attrition. All analyses and the randomization sequence were conducted using R."		
**12a-i) Imputation techniques to deal with attrition / missing values**		
We did not impute in our study		
**12b) CONSORT: Methods for additional analyses, such as subgroup analyses and adjusted analyses**		
We did not do subgroup analysis		
RESULTS		
**13a) CONSORT: For each group, the numbers of participants who were randomly assigned, received intended treatment, and were analysed for the primary outcome**		
"All clinicians at the MDC agreed to take part, including the regularly scheduled staff (n=14) as well as rotating residents, fellows, and ad hoc specialists (total clinicians n=34). We approached 30 patients to meet our targeted sample of 24 participants (80% recruitment rate; 22 through e-consent and 2 through in-person consent; CONSORT flow diagram, Multimedia Appendix 2). In TAU, demographic information for 2 patients was unavailable: one patient entered hospice care before their first visit, while another did not receive the demographic questionnaire. About 65% (14/22) were male patients with a median age of 66.5 years (Table 1). Of the 12 participants in the intervention arm, study assessments were completed by 11 at 1 week and 7 participants at 3 months. Of the 12 participants in TAU, study assessments were completed by 9 at 1 week and by 5 participants at 3 months. More information is given in Multimedia Appendix 2. Attrition was primarily due to death (n=6 in TAU and n=4 in the intervention arm), while one patient was dissatisfied with their allocation to TAU and one newly diagnosed patient withdrew to focus on managing their condition. We recruited 100% of caregivers approached (n=21 caregivers), with 12 in the intervention arm and 9 in TAU. About 70% (14/21) were female caregivers, with a median age of 58.5 years (Table 1). After patients passed away, caregivers were not contacted further."		
**13b) CONSORT: For each group, losses and exclusions after randomisation, together with reasons**		
"Attrition was primarily due to death (n=6 in TAU and n=4 in the intervention arm), while one patient was dissatisfied with their allocation to TAU and one newly diagnosed patient withdrew to focus on managing their condition."		
**13b-i) Attrition diagram**		
Yes, multimedia appendix 2 has the consort flow		
**14a) CONSORT: Dates defining the periods of recruitment and follow-up**		
"Recruitment began in April 2021 and continued through March 2022"		
**14a-i) Indicate if critical "secular events" fell into the study period**		
Not applicable to our study		
**14b) CONSORT: Why the trial ended or was stopped (early)**		
"Data collection concluded in June 2022."		
**15) CONSORT: A table showing baseline demographic and clinical characteristics for each group**		
This information is included in Table 1		
**15-i) Report demographics associated with digital divide issues**		
This information is included in Table 1		
**16a) CONSORT: For each group, number of participants (denominator) included in each analysis and whether the analysis was by original assigned groups**		
**16-i) Report multiple "denominators" and provide definitions**		
"A total of 144 unique encounters with clinicians were recorded, for approximately 12 recordings per patient in the intervention arm. Of patients receiving recordings, 75% (9/12) viewed at least one video, with an average of 6 videos viewed each. One participant did not think they required videos, one patient did not want to watch the videos, and one patient's condition progressed quickly, and they did not feel the videos were relevant. Of the 55 videos watched, 36% (n=20) were in neurology, and 29% (n=16) were in physical and occupational therapy. Of note, 3 caregivers continued watching videos after patients had passed away."		
**16-ii) Primary analysis should be intent-to-treat**		
"Mean intervention feasibility (4, range 2-5, 95% CI 3.05-4.7) and acceptability (4, range 2-5, 95% CI 2.79-4.83) of intervention measures were reported by patients in the intervention arm."		
**17a) CONSORT: For each primary and secondary outcome, results for each group, and the estimated effect size and its precision (such as 95% confidence interval)**		
This is addressed in Table 2		
**17a-i) Presentation of process outcomes such as metrics of use and intensity of use**		
We did not have process outcomes in this study		
**17b) CONSORT: For binary outcomes, presentation of both absolute and relative effect sizes is recommended**		
Our outcomes were all continuous		
**18) CONSORT: Results of any other analyses performed, including subgroup analyses and adjusted analyses, distinguishing pre-specified from exploratory**		
Table 3 includes qualitative outcomes		
**18-i) Subgroup analysis of comparing only users**		
We did not perform a subgroup analysis of only users		
**19) CONSORT: All important harms or unintended effects in each group**		
"The utility of clinic visit recordings as mementos or their effects on bereavement is similarly less understood; videos have been identified as valuable by caregivers after patients have passed away, but the identified videos included home movies and personal videos rather than those occurring in a medical encounter"		
**19-i) Include privacy breaches, technical problems**		
"The utility of clinic visit recordings as mementos or their effects on bereavement is similarly less understood; videos have been identified as valuable by caregivers after patients have passed away, but the identified videos included home movies and personal videos rather than those occurring in a medical encounter [37]."		
**19-ii) Include qualitative feedback from participants or observations from staff/researchers**		
See the "qualitative results" section and "table 3"		
DISCUSSION		
**20) CONSORT: Trial limitations, addressing sources of potential bias, imprecision, multiplicity of analyses**		
**20-i) Typical limitations in ehealth trials**		
See the "Limitations" section		
**21) CONSORT: Generalisability (external validity, applicability) of the trial findings**		
**21-i) Generalizability to other populations**		
See the "Comparison with Previous Work" section		
**21-ii) Discuss if there were elements in the RCT that would be different in a routine application setting**		
Our trial took place in the setting of a routine application		
**22) CONSORT: Interpretation consistent with results, balancing benefits and harms, and considering other relevant evidence**		
**22-i) Restate study questions and summarize the answers suggested by the data, starting with primary outcomes and process outcomes (use)**		
"In our sample, patients, caregivers, and clinicians found video recording to be highly acceptable and feasible to implement at ALS MDCs. While we experienced attrition, we found recordings were used by patients and their caregivers to remember what was said in visits and to share information from visits with family members."		
**22-ii) Highlight unanswered new questions, suggest future research**		
"Future work could explore the impacts of recording on caregiver outcomes identified in the scoping review, including resilience and coping. The utility of clinic visit recordings as mementos or their effects on bereavement is similarly less understood; videos have been identified as valuable by caregivers after patients have passed away, but the identified videos included home movies and personal videos rather than those occurring in a medical encounter [37]."		
Other information		

**23) CONSORT: Registration number and name of trial registry**		
"ClinicalTrials.gov NCT04719403; https://clinicaltrials.gov/study/NCT04719403"		
**24) CONSORT: Where the full trial protocol can be accessed, if available**		
See "Multimedia Appendix 1"		
**25) CONSORT: Sources of funding and other support (such as supply of drugs), role of funders**		
"The study team would like to gratefully acknowledge the financial support of the Diamond Endowment at the Dartmouth-Hitchcock Department of Neurology."		
**X26-i) Comment on ethics committee approval**		
"This study received ethical approval from the Dartmouth Health Institutional Review Board (02000798) and was registered on ClinicalTrials.gov (NCT04719403) in January 2021"		
**x26-ii) Outline informed consent procedures**		
"Participants were consented to according to Dartmouth Health protocols and were given paper and electronic copies of consent forms for review."		
**X26-iii) Safety and security procedures**		
This can be found in the clinicaltrials.gov posting		
**X27-i) State the relation of the study team towards the system being evaluated**		
We had no conflicts to declare.		
